# Therapeutic Potential of Green-Engineered ZnO Nanoparticles on Rotenone-Exposed *D. melanogaster* (Oregon R^+^): Unveiling Ameliorated Biochemical, Cellular, and Behavioral Parameters

**DOI:** 10.3390/antiox12091679

**Published:** 2023-08-28

**Authors:** Shabnam Shabir, Amit Sehgal, Joydeep Dutta, Inderpal Devgon, Sandeep K. Singh, Walaa F. Alsanie, Abdulhakeem S. Alamri, Majid Alhomrani, Abdulaziz Alsharif, Mohammed Abubaker Mohammed Basalamah, Hani Faidah, Farkad Bantun, Abdullah Ali Saati, Emanuel Vamanu, Mahendra P. Singh

**Affiliations:** 1 School of Bioengineering and Biosciences, Lovely Professional University, Phagwara 144411, Punjab, India; 2 Indian Scientific Education and Technology Foundation, Lucknow 226002, Uttar Pradesh, India; 3 Department of Clinical Laboratory Sciences, The Faculty of Applied Medical Sciences, Taif University, Taif 21944, Saudi Arabia; 4 Centre of Biomedical Sciences Research (CBSR), Deanship of Scientific Research, Taif University, Taif 21944, Saudi Arabia; 5 Department of Pathology, Faculty of Medicine, Umm Al-Qura University, Makkah 24382, Saudi Arabia; 6 Department of Microbiology, Faculty of Medicine, Umm Al-Qura University, Makkah 24382, Saudi Arabia; 7 Department of Community Medicine & Pilgrims Healthcare, Faculty of Medicine, Umm Al-Qura University, Makkah 24382, Saudi Arabia; 8 Faculty of Biotechnology, University of Agricultural Sciences and Veterinary Medicine, 011464 Bucharest, Romania; 9 Department of Zoology and Centre of Genomics and Bioinformatics, DDU Gorakhpur University, Gorakhpur 273009, Uttar Pradesh, India

**Keywords:** zinc oxide nanoparticles, antioxidants, antibacterial agents, medicinal plants, green synthesis, oxidative stress

## Abstract

Nanotechnology holds significant ameliorative potential against neurodegenerative diseases, as it can protect the therapeutic substance and allow for its sustained release. In this study, the reducing and capping agents of *Urtica dioica* (UD), *Matricaria chamomilla* (MC), and *Murraya koenigii* (MK) extracts were used to synthesize bio-mediated zinc oxide nanoparticles (ZnO-NPs) against bacteria (*Staphylococcus aureus* and *Escherichia coli*) and against rotenone-induced toxicities in *D. melanogaster* for the first time. Their optical and structural properties were analyzed via FT-IR, DLS, XRD, EDS, SEM, UV–Vis, and zeta potential. The antioxidant and antimicrobial properties of the fabricated ZnO-NPs were evaluated employing cell-free models (DPPH and ABTS) and the well diffusion method, respectively. Rotenone (500 µM) was administered to *Drosophila* third instar larvae and freshly emerged flies for 24–120 h, either alone or in combination with plant extracts (UD, MC, an MK) and their biogenic ZnO-NPs. A comparative study on the protective effects of synthesized NPs was undertaken against rotenone-induced neurotoxic, cytotoxic, and behavioral alterations using an acetylcholinesterase inhibition assay, dye exclusion test, and locomotor parameters. The findings revealed that among the plant-derived ZnO-NPs, MK-ZnO NPs exhibit strong antimicrobial and antioxidant activities, followed by UD-ZnO NPs and MC-ZnO NPs. In this regard, ethno-nano medicinal therapeutic uses mimic similar effects in *D. melanogaster* by suppressing oxidative stress by restoring biochemical parameters (AchE and proteotoxicity activity) and lower cellular toxicity. These findings suggest that green-engineered ZnO-NPs have the potential to significantly enhance outcomes, with the promise of effective therapies for neurodegeneration, and could be used as a great alternative for clinical development.

## 1. Introduction

Homeostasis among both antioxidants and oxidants regulates the redox status of cells. Any imbalance between these two processes that can result in necrosis or apoptosis determines the oxidative status of the cell [1]. The key factor contributing to the increased oxidative stress sensitivity of brain cells is reactive oxygen species (ROS). Although oxygen (O_2_) is typically a nonreactive molecule, it can be metabolized to form oxidation products such as superoxide anions (O_2_^−^), hydroxyl radicals (OH^−^), and several other reactive species in the body. It leads to dreaded conditions, including neurological diseases [2]. Cellular defense systems that include endogenous antioxidants such as catalase, glutathione reductase, superoxide dismutase, glutathione, and glutathione peroxidase contribute to detoxification [3]. Significant cell biomolecules such as lipids, DNA, and proteins are damaged by the degradation of these defense mechanisms, which may lead to alterations in cell signaling pathways [4].

One of the great challenges of sending drugs to the central nervous system (CNS) is posed by the presence of blood–brain barrier (BBB) which is a tight barrier and highly selective in allowing molecules to enter the CNS [5]. Systemic administration of drugs to the CNS is a substantial obstacle, owing to their short half-life, considerable first-pass digestion, restricted accessibility to the brain, and potential adverse impact when accessing nontarget peripheral organs [6]. As a result, developing systemic delivery mechanisms with higher potency is critical for CNS pharmacotherapy. Cholinesterase (ChE) inhibitors, tacrine, N-methyl-D-aspartate (NMDA) agonists in connection with vitamin D, levodopa, or other dopaminergic agonists and memantine have never been utilized in conventional medicine or therapeutics to alleviate anything apart from motor symptoms by replenishing neurotransmitters [7]. However, prolonged use of these medications can have substantial side effects, including other motor challenges [8]. To stop or reduce the progression of numerous neurodegenerative disorders, researchers must create new natural neuroprotective agents due to the inadequacy of treatment medicines [9]. By activating the Nrf2/Akt/PI3K cascade and eliminating free radicals, nutraceuticals and other phytonutrients have been proven to have protective benefits and to reduce the consequences of neurodegenerative conditions [10].

The World Health Organization (WHO) estimates that 78% of people worldwide currently use phytomedicine as their primary source of treatment [11]. The Ayurvedic medicinal system has long employed the plants UD (*Urtica dioica*), MC (*Matricaria chamomilla*), and MK (*Murraya koenigii*) as well-known nerve relaxants and cognition boosters [12,13]. These three herbs include phytochemicals (quercetin, alkaloids, vitamins, and polyphenols) with proven protective properties against rotenone-induced toxicities in *Drosophila melanogaster* as reported in our previous study [14]. Drug targeting and delivery of dietary antioxidants to the brain face significant obstacles in treating oxidative stress-related disorders due to the BBB, particularly neurodegenerative diseases. Novel approaches for the improved crossing of the BBB must be developed to progress in effective therapies [15]. The use of novel nanotechnology-based methods, such as nanoparticles as nanocarriers, may cross the blood–brain barrier and deliver the proper dosage of drug/medicine to the target brain region [16].

**Figure 1:**
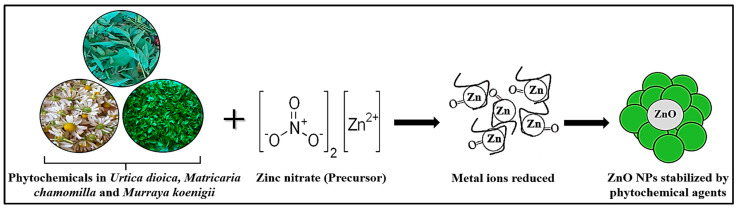
Mechanism of fabricating ZnO nanoparticles by using *U. dioica*, *M. chamomilla*, and *M. koenigii* extracts.

Nanotechnology is a novel and rapidly evolving technique with several potential applications. It entails the fabrication and use of substances having one or more dimensions ranging from 1 to 100 nm [17]. Using plant extracts from various plant species, many metals, including silver (Ag), zinc oxide (ZnO), gold (Au), and many others, have been used in the biosynthesis of NPs [18,19]. Scientific research often involves exploring new materials and their properties. Choosing ZnO nanoparticles over Au nanoparticles might stem from a desire to investigate lesser-studied materials or to discover unique characteristics that could lead to innovative applications. The high pharmacological qualities of zinc oxide have enhanced its usage in healthcare applications, such as antioxidant, antibacterial, and anticancer activities [20]. Furthermore, zinc oxide, along with four other compounds of zinc, has been designated as generally recognized as safe (GRAS) by the US-FDA “Food and Drug Administration” [21]. Although UD [22], MC [23], and MK [24] NPs have been utilized in several in vitro and in vivo studies, there is currently less information available regarding the beneficial impact of these NPs on cellular and neurological issues. The reaction mechanism in Figure 1 shows that zinc oxide nanoparticles (ZnO-NPs) can be fabricated through the reduction of Zn^+2^ using plant fractions.

This work used the green method to fabricate ZnO-NPs from the UD, MC, and MK fractions. The phyto-synthesized ZnO-NPs were analyzed using FT-IR, SEM, UV–Vis, zeta potential, X-ray diffraction techniques, energy dispersive X-rays, and dynamic light scattering. The well diffusion approach was used to investigate the antimicrobial activities of biosynthesized ZnO-NPs against *Staphylococcus aureus* and *Escherichia coli*. Moreover, we aimed to explore the therapeutic potential of phytofabricated ZnO nanoparticles against the commonly used neurotoxic organic pesticide rotenone in vivo in *Drosophila* (Oregon R^+^) for the first time. We also evaluated its behavioral parameters on *Drosophila*, “which has previously been proven to be a valuable indicator for neurodegenerative pathologies such as PD (Parkinson’s disease)”.

## 2. Materials and Methods

### 2.1. Reagents and Chemicals

Zinc nitrate hexahydrate, bovine serum albumin, and acetylthiocholine iodide were purchased from Sigma (Roeder-mark, Germany). Ellman’s reagent (C_14_H_8_N_2_O_8_S_2_), “1, _1_diphenyl-2-picrylhydrazyl” (C_18_H_12_N_5_O_6_), Folin–Ciocalteu’s reagent (C_6_H_6_O), ABTS (C_18_H_18_N_4_O_6_S_4_), gallic acid (C_7_H_6_O_5_), aluminum chloride hexahydrate (AlCl_3_ 6H_2_O), ascorbic acid (C_6_H_8_O_6_), sulfuric acid (H_2_SO_4_), ferric chloride (FeCl_3_), ethanol, nutrient agar, sodium benzoate (C_7_H_5_NaO_2_), and sodium carbonate (Na_2_CO_3_) were acquired from Hi-Media (Mumbai, India). Calorimetric measurements were carried out using an ultraviolet–visible spectrometer (Shimadzu UV-1601, Tokyo, Japan). All glassware was rinsed with deionized water and dried in an oven before usage.

### 2.2. Harvesting and Identification of Plant Materials

Fresh *U. dioica* (HBJU-17038) leaves were obtained from local apple orchards and kitchen gardens of Sopore (Sopore, J&K, India) before the plants began to generate seeds. The Mediaroma Agro Company Ltd. (Kaskanj, UP, India) provided the *Matricaria chamomilla* (HBJU-17039) flowers, while *Murraya koenigii* (HBJU-17040) young leaves were acquired from Ayushya Vatika of the LPU Campus (Punjab, India). “A taxonomist from the department of botany at the University of Jammu (Jammu, India) verified the identification and certification of the therapeutic plants”.

#### 2.2.1. Extraction of Plants

The freshly obtained plant flowers or leaves were cleaned with distilled water to eliminate surface pollutants before being left to shade dry for a week. Plant material was dried, crushed into fine powder utilizing a grinder and then sieved to obtain the desired particle size. A 10% plant fraction was made by steeping 5 g (±0.05) of plant powder in deionized water (50 mL) for 15 min at 95–100 °C [25]. The plant fractions were then filtered using Whatman No. 1 filter paper. The extract from UD and MK leaves was dark green, although the fraction from MC flowers was pale yellow. After that, it was centrifuged at 2500 rpm for 5 min to remove the debris, and the resultant supernatant was kept for further analysis at 4 °C.

#### 2.2.2. Biosynthesis of ZnO Nanoparticles

To synthesize ZnO-NPs for the experiment, a modified green synthesis technique was used from a previous study [26]. Aqueous plant extract (UD, MC, and MK) (25 mL) was combined/mixed with 0.3 mM Zn(NO_3_)_2_.6H_2_O solution by constant stirring. The mixture was then treated with 2 M NaOH to maintain the pH at 12 (improved biocompatibility and reduced toxicity). The reaction was stirred for two hours at 65 °C using a magnetic stirrer. After that, the mixture was collected and heated in an oven at a constant temperature overnight until it formed a dense golden/yellow paste. This yellow paste was then thoroughly dried and calcined for 2 h at 400 °C. Subsequently, a pale white powder of ZnO nanoparticles was obtained and used for further characterization. The calcination process is temperature-dependent and eliminates impurities from the sample to yield a pure form of the nanoparticle.

This process was repeated at two different temperatures (75 °C and 55 °C), which were both above and below the optimum temperature. The reaction between Zn(NO_3_)_2_.6H_2_O and plant extracts did not take place at 55 °C, which resulted in the formation of nano ZnO. Moreover, the high temperature generated ashes when 75 °C was used as the operating temperature. However, a yellow color change was detected with no negative effects at 65 °C. Furthermore, the sample was proven to be ZnO-NPs throughout the characterization process. As a result, temperature has a momentous effect on the generation of nanoparticles. The obtained light-yellow fine powder was employed for morphological, antioxidant, and antimicrobial properties.

#### 2.2.3. Characterization of Synthesized ZnO-NPs Using Microscopy Techniques

Microscopy techniques are an essential tool for characterization that provide precise descriptions of the shape, structure, size, and chemical composition of zinc oxide NPs. The following microscopy techniques were used for the structural, morphological, and molecular identification of ZnO-NPs.

#### 2.2.4. UV–Visible Analysis

The optical properties of phyto-fabricated ZnO-NPs of selected plants were observed using the absorption spectrum of NPs [27,28]. This was recorded with an ultraviolet–visible spectrometer (Shimadzu UV-1601, Tokyo, Japan) with a wavelength range of 200–800 nm. The SPR “surface plasmon resonance” was the result of the light interaction with the moving surface electrons of ZnO nanoparticles. At room temperature, the UV–Vis spectrum was recorded with a path length of 1 cm in a quartz cuvette.

#### 2.2.5. Powder X-ray Diffraction (XRD)

The XRD technique was utilized to determine the crystalline size, stress, and crystalline phase identification of ZnO nanoparticles [29]. XRD analyses were performed using a diffractometer (Bruker D8 Advance) employing a Cu K_α1_ beam (λ = 1.54060 Ǻ, 30 mA, 45 kV). The diffractograms were obtained in the range of 20–90° at 2q with a scan period of 2 s per step and a step size of 0.05°. During XRD analysis, synthesized UD-ZnO, MC-ZnO, and MK-ZnO NPs were subjected to intense XRD rays that penetrated through them and provided important structural information. The nano size is indicated by the widening of the XRD pattern. The average diameter of ZnO nanoparticles is estimated using the Debye–Scherer equation:

*d* = k λ/βcos θ

where *d* is the average crystallite size (measured in nanometers), k is the crystallite form factor (which is approximately 0.9), λ is the X-ray wavelength (1.54060 Ǻ), β is the full width at half maximum (FWHM) of the X-ray diffraction peak (measured in radians), and θ is the Bragg angle. The relationship below can be used to calculate the lattice parameters.

1/d^2^ = 4/3 [h^2^ + hk + l^2^/a^2^] + l^2^/c^2^

#### 2.2.6. Fourier Transform Infrared Spectroscopy

FT-IR is an efficient technique for detecting functional groups, molecular structures, and chemical bonds in compounds [30]. As seen in the spectra, the light wavelength absorbed reveals the coordination complex. The infrared absorption spectrum of a substance reveals the structure of its chemical bonds [31]. To detect the functional groups involved in the fabrication of biogenic ZnO-NPs, a Perkin-Elmer spectrophotometer was used. A 10 mg ZnO-NP sample was contained in a 100 mg KBr pellet to generate a translucent sample disc. The ZnO-NPs from each plant material were then submitted to an FT-IR spectrophotometer. The biosynthesized ZnO-NPs were analyzed in the infrared band at room temperature, with a 4 cm^−1^ resolution and frequency range of 400–4000 cm^−1^.

#### 2.2.7. Zeta Potentiometer

The determination of zeta potential is a significant technique with nanocrystals for estimating surface charge in colloidal solution [32]. The stability of the synthesized ZnO-NPs of selected plants was analyzed by using a particle size and zeta potential analyzer (Malvern Zeta sizer Nano ZS90). Nanocrystals with a large negative or positive zeta potential indicate strong physical durability of NPs due to electrostatic repulsion of subatomic particles [33].

#### 2.2.8. Dynamic Light Scattering (DLS) Analysis

The particle size was determined using the Nano plus, Micromeritics (USA) DLS technique. The apparatus is sensitive to molecular weights as low as 250 Da and is capable of detecting compounds dispersed in liquids with particle sizes ranging from 0.1 nm to 12.2 m and concentrations of 0.00001 to 40% [34]. By analyzing the angles at which an incoming beam of light was dispersed as a function of the Brownian motion of the colloidal particles, the size of the NPs (multimodal size distribution) was evaluated.

#### 2.2.9. Scanning Electron Microscopy Analysis

SEM is one of the most commonly used techniques for evaluating NPs. Electron-sample interactions provide signals that reveal information about the sample, such as its surface shape and chemical makeup [35]. The morphology of the synthesized ZnO-NPs of selected plants (UD, MC, and MK) was studied using a JEOL JSM-7610F Plus with an Au sputter coater. The samples were coated with a thin coating of gold or gold–palladium alloy to improve secondary electron emission and avoid surface charge so that the specimen conducted uniformly and provided a homogenous surface for study and imaging.

#### 2.2.10. Energy-Dispersive X-ray Spectroscopy

The presence of elemental zinc and oxygen was detected using an OXFORD EDS LN2 [36]. The samples were dried at room temperature before being tested for the composition of the biogenic zinc oxide NPs. The EDX spectra of the “ZnO-NPs” that were synthesized using plant extracts (UD, MC, and MK) demonstrate their purity and composition.

#### 2.2.11. DPPH Free Radical Scavenging Activity

The antioxidant activity of biosynthesized ZnO-NPs from UD, MC, and MK, as well as their extracts, was estimated by using a 1,1-diphenyl-2-picrylhydrazyl (DPPH) assay [37]. A fresh DPPH stock mixture was prepared by dissolving 11 mg of DPPH in methanol (50 mL). The DPPH stock mixture was further diluted with the addition of methanol to attain an optical density range of 0.8 to 1.0. Each sample (0.5, 1.0, and 1.5 mg/mL) was fused with 2 mL of DPPH mixture. A spectrophotometer (Shimadzu UV-1601, Tokyo, Japan) was utilized to detect the absorbance at 517 nm after incubation for 30 min. Methanol was utilized as a control, and the DPPH mixture was utilized as a blank. Ascorbic acid was used as the standard reference compound. The lower the absorbance, the stronger the free radical activity of the reaction mixture. Experiments were carried out in triplicate. The % DPPH scavenging effect was computed using the equation below:

Inhibition of DPPH (%) = [(A_control_ − A_sample_)/A_control_] × 100

where A_sample_ is the absorbance of biosynthesized ZnO-NPs from UD, MC, and MK, as well as their extracts, and A_control_ is the absorbance of the DPPH mixture.

#### 2.2.12. ABTS Free Radical Scavenging Activity

The scavenging ability of biosynthesized ZnO nanoparticles from UD, MC, and MK, as well as their extracts, was evaluated using the ABTS “2,2′-azino-bis(3-ethylbenzothiazoline-6-sulfonic acid” test [38]. A radical cation of ABTS stock solution/mixture was formed by dissolving 36 mg of ABTS in methanol (10 mL) in a 1:1 ratio with 57 mg of potassium persulfate in methanol (10 mL). The solution was then kept for 16 h at ambient temperature in the dark. The ABTS stock solution was further diluted with the addition of methanol to attain an optical density range of 0.8 to 1.0. Each sample (0.5, 1.0, and 1.5 mg/mL) was mixed with ABTS solution (2 mL). A spectrophotometer was used to measure the absorbance (745 nm) after 30 min of incubation. Experiments were carried out in triplicate. The % ABTS scavenging effect was computed using the equation below:

Inhibition of ABTS (%) = [(A_control_ − A_sample_)/A_control_] × 100

where A_sample_ is the absorbance of biosynthesized ZnO-NPs from UD, MC, and MK, as well as their extracts, and A_control_ is the absorbance of ABTS stock solution.

#### 2.2.13. EC_50_ (Dose–Response Relationship)

An EC_50_ is a numerical analysis of the percentage of an antibody, drug, or poisonous chemical that, after a certain amount of time, causes a reaction that is half as strong as the peak response. Data analyses for dose–response experiments and free radical scavenging activity were performed by using CompuSyn software (Version 1.0) to measure the potential of biosynthesized ZnO-NPs from UD, MC, and MK, as well as their extracts. Higher radical scavenging activity is predicted by lower EC_50_ values [39].

### 2.3. Microorganisms and Fly Strain

Bacterial strains of “*Escherichia coli* and *Staphylococcus aureus*” were cultured in a sterile environment at the Lovely Professional University (Punjab, India). The Oregon R^+^ strain of *D. melanogaster* (wild type) was fed a standard diet including yeast, agar, maize flour, propionic acid, sodium benzoate, and sulfur-free sugar. The flies were sustained at 24 ± 1 °C, 12-h dark/light cycle, and 65–70% relative humidity [40].

#### 2.3.1. Antimicrobial Activity

The antimicrobial potential of green-engineered ZnO nanoparticles was evaluated against bacteria, including *Staphylococcus aureus* and *Escherichia coli*. The bacteria were cultured on nutritional agar medium for 24 h at 37 °C [41]. One colony of bacteria was selected and suspended in 5 mL of physiological serum using a sterile inoculating loop. The turbidity of the bacterial isolates was adjusted to match a McFarland criterion of 0.5. Inoculum tubes were swabbed with sterile swabs. By streaking the swabs across Mueller–Hinton agar plates, bacteria were inoculated. The well diffusion method was used for the antibacterial test. Each petri plate had three wells (3 mm in diameter) that were filled with a negative control (deionized water), a positive control (chloramphenicol), and 100 µg of ZnO nanoparticles. The zone of inhibition on the plates was measured as microbial species inhibition [42].

#### 2.3.2. Biosynthesized ZnO-NP Concentration and Rotenone Exposure

A series of dosages (0.01, 0.025, 0.05, and 0.1%) of biosynthesized ZnO-NPs were tested in preliminary studies using small numbers of *Drosophila* flies to determine whether pretreatment had any effect on fly survival during the trial. One concentration was chosen as the optimal concentration; it was 0.1% per unit of medium. Furthermore, UD-ZnO, MC-ZnO, and MK-ZnO NPs were tested for their cellular and neuroprotective abilities using rotenone (ROT) at a concentration of 500 µM, which was chosen based on our earlier study of *D. melanogaster* and those of other previous reports [43,44,45].

#### 2.3.3. Experimental Design

The flies/larvae (third instar) of *D. melanogaster* were segregated into 6 groups for the experiment. Group I received the standard *Drosophila* larval diet as a control, while Group II received food that had been combined with DMSO (0.1% vehicular control). In Group III, 500 µM ROT was used alone. UD-ZnO (0.1%), MC-ZnO (0.1%), and MK-ZnO (0.1%) NPs added to Group IV, Group V, and Group VI, respectively, were co-exposed to ROT. Larvae were allowed to eat standard food or food that had been treated for 24 or 48 h, respectively, with ROT or ROT + ZnO-NPs of UD, MC, or MK. The ability of the flies to climb and jump was evaluated after 120 h (5 days) of exposure. We evaluated the modulatory effects of biosynthesized ZnO-NPs on rotenone-induced acetylcholinesterase inhibition, locomotor dysfunction, lethality, cellular toxicity, and proteotoxicity.

#### 2.3.4. Dye Exclusion (Trypan Blue) Assay

A dye exclusion test was used to evaluate cell viability with minor modifications as reported by Krebs and Feder (1997) [46]. The distinction between living and dead tissue cells can be detected using this quick and easy method. The entire larval body and gut are used to assess cell death. It is based on the membrane impermeability of blue dye; live cells have an intact cell membrane, and trypan blue cannot pass through the cell membrane and enter the cytoplasm. After treatment, by using phosphate buffer saline (0.1 M, pH 7.4), 10–12 larvae were washed three times. Thereafter, the dissected midguts or whole larvae were immersed in trypan blue dye solution (0.4%) for 15 min. A stereomicroscope was used to examine the larvae; pictures were taken for trypan blue dye exclusion grading and thoroughly scrutinized.

#### 2.3.5. Homogenate Preparation

Third instar *Drosophila* larvae from the DMSO, control (untreated/normal), ROT, and ROT with biosynthesized ZnO-NP-treated groups were dissected and homogenized in ice-cold phosphate buffer (pH 7.4, 0.1 M) with KCl (0.15 M) to generate 10% homogenate/cytosol. Samples were homogenized and then centrifuged (10,000× *g*) for 10 min at 4 °C. The supernatant was filtered using a nylon mesh sieve with particles no larger than 10 mm, and it was then employed in a number of experiments [47].

#### 2.3.6. Acetylcholinesterase (AChE) Enzymatic Assay

The previously demonstrated method [48], with a few minor modifications [49], was used to measure the activity of acetylcholinesterase in the brain tissues of *Drosophila* flies. Briefly, head regions from untreated and treated flies were homogenized at 10% in 50 mmol L^−1^ HEPES buffer with protease inhibitor and then centrifuged (10,000× *g*) for 15 min. The test mixture included phosphate buffer, acetylthiocholine iodide as the substrate, DTNB (5,5-dithiobis(2-nitrobenzoic) acid), and tissue homogenate. The rate of acetylthiocholine iodide breakdown was calculated at a wavelength of 412 nm, and the findings were reported as mol min^−1^ mg^−1^ protein.

#### 2.3.7. Protein Estimation Assay

The total protein content of *Drosophila melanogaster* midgut homogenate was determined using the Bradford (1976) procedure with bovine serum albumin (BSA) as the standard protein [50].

#### 2.3.8. Jumping Assay

To evaluate neuromuscular function, a jumping activity test was conducted. Jumping activity appears to be controlled by the rate of motor function [51]. The distance jumped by newly emerged flies was measured starting from the bottom of the vial after each fly was moved one at a time to a vial labelled 1–12 cm. The jumping ability was evaluated by averaging the number of jumps over five duplicates. Each group consisted of five replicates and contained 100 flies.

#### 2.3.9. Negative Geotaxis (Climbing Assay)

Anaesthetized test flies were kept in a vertical glass vial that was approximately 25 cm in length and 1.5 cm in diameter [52]. The flies were firmly tapped to the bottom of the vial after a short rest period. After one minute, flies that climbed to the top of the vial and those that stayed at the base were counted differently. Every 60 s, the data were expressed as a percentage of flies that eluded capture beyond a minimal distance of 6 cm. Each replication of the experiment used twenty adults. “The experiment was performed in triplicate”, and the average of the three trials for each fly group, including the control, was used to determine the findings for each replication. For each trial, a PI (performance index) was computed and presented as follows:

1/2[(n_tot_ + n_top_ − n_bot_)/n_tot_]

#### 2.3.10. Memory Assay

This test was performed on the flies after raising them in the conditions that were utilized as a learning condition to perform the memory assay [53]. Take 10 flies in each vial in triplicate for the assay (total of 30 flies) in every group. Transfer the flies for half an hour in the food + dark and no food + light conditions for eight hours. Flies were maintained in light after experiencing the conditions. These constraints were imposed for three days. Flies were deprived for an hour on the fourth day before the analysis began. These constraints were imposed for a period of three days. Flies were deprived for an hour on the fourth day before beginning the analysis. After one hour, food is placed on one side of the T-maze that is dark, and no food on the other side that is bright, and the number of times the fly will avoid or follow the taught circumstances will be recorded. The experiment was not performed immediately since learning was not assessed, but rather for memory conditions influenced by neurodegenerative disorders. Examine the number of flies that arrive at the T and go to the designated sides. For the remaining one minute, repeat the procedure 10 times with the same vial. Record the number of flies as the percentage of the total number of flies that advances towards light and dark. The experiment was performed 5 times separately.

#### 2.3.11. Larval Crawling Assay

Larval crawling behavior is a basic experiment for understanding the rhythmic behavior of larvae and detecting any neurological abnormalities [54]. Third instar larvae of *Drosophila* were used to record crawling behavior. To clear the cuticular dietary substances, larvae were rinsed in phosphate-buffered saline (PBS). The larvae were allowed to crawl on 2% agar plates prepared for the larval crawling experiment. Graph paper was placed beneath the plate to record the distance travelled by the larva. The larval trailing route was noted, and crawling speed was determined based on the time it took each larva to reach the periphery of the agar plate. The speed was measured in millimeters per second on average.

### 2.4. Statistical Analysis

Colorimetric data reported as the mean ± SD and *n* = 3 were evaluated for significant differences in SPSS statistical analysis software (Version 18) utilizing two-way ANOVA and Tukey’s test. The significance was assessed using *p* < 0.05.

## 3. Results

The subsections below provide the results of the characterization of UD-ZnO, MC-ZnO, and MK-ZnO NPs. Moreover, their antioxidant, antimicrobial, and ameliorative potential against “rotenone-induced proteotoxicity, locomotor dysfunctions, lethality, acetylcholinesterase inhibition, and cellular toxicity was undertaken”.

### 3.1. Analytical Assays

#### 3.1.1. Plant-Based Extraction Mechanism of ZnO Nanoparticle Synthesis

**Figure 2:**
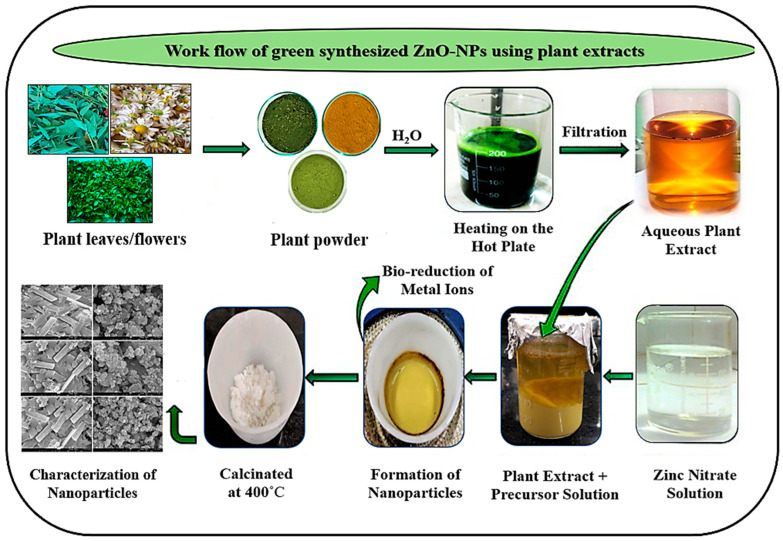
The plant-mediated mechanism of biogenic ZnO nanoparticles includes the extraction of the plant fraction, combining of the metal precursor solution in the plant fraction, and generation of green-engineered NPs. The NPs were then analyzed using techniques such as FTIR, SEM, XRD, EDX, and UV–Vis.

The green fabrication mechanism for the generation of ZnO nanoparticles based on plant phytochemicals can function via stabilizing and capping compounds to convert metal salts to metal nanoparticles. The major phytochemicals, including alkaloids, aldehydes, terpenoids, flavonoids, and phenolic acids, served as stabilizing agents that prevented particle agglomeration. These phytoconstituents (capping/reducing agents) are present in various plant extracts at varying concentrations. Thus, the synthesis of NPs is considerably influenced by the composition of the plant fraction. Zinc (II) ions in plant extracts are converted to metallic zinc instead of generating a coordinated complex. Following the full reduction in the zinc precursor, metallic zinc and the dissolved oxygen in the solution reacted, resulting in the generation of zinc oxide nuclei. The fabrication, stability, and generation of NPs are all dependent on factors including pH, metal salt content, contact time, temperature, and phytochemical composition of the plant fraction (Figure 2). 

#### 3.1.2. Characterization of Biosynthesized Zinc Oxide Nanoparticles (ZnO-NPs)

Characterization is a fundamental process for determining the size, morphology, shape, size distribution, composition, surface area, and surface charge of green fabricated ZnO-NPs. The techniques that have been employed to characterize ZnO-NPs are listed below.

#### 3.1.3. Optical Properties of ZnO-NPs Using UV–Vis Spectroscopy

**Figure 3:**
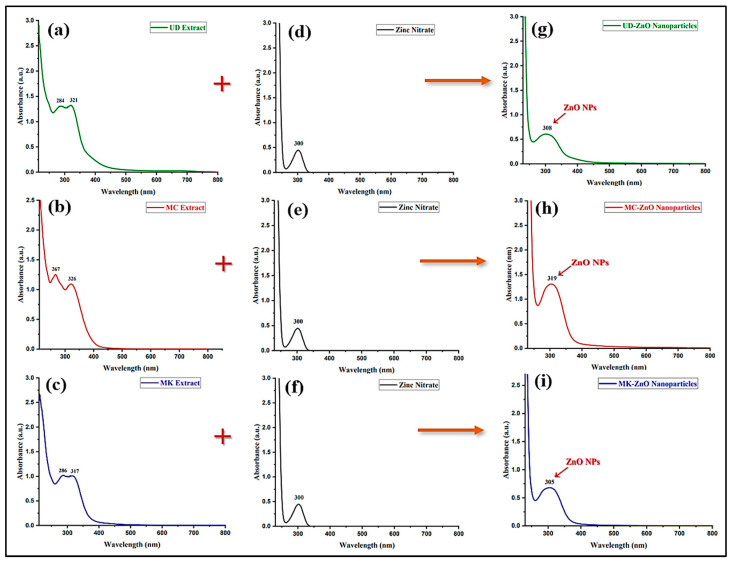
UV–Visible spectra of zinc nitrate (**d**–**f**), *U. dioica* (UD), *M. chamomilla* (MC), and *M. koenigii* (MK) extracts (**a**–**c**) and their respective phytofabricated ZnO-NPs (**g**–**i**). + represents addition of plant extracts into zinc nitrate solution and → represents synthesis of zinc oxide nanoparticles.

The optical attributes of the synthesized ZnO nanoparticles were assessed utilizing ultraviolet and visible absorption spectroscopy. The absorbance spectra between 200 and 800 nm were employed to record the biosynthesis of UD-ZnO, MC-ZnO, and MK-ZnO NPs in an aqueous solution. In the absence of a precursor, the plant fraction did not change color; however, the leaf extract became dark yellow when zinc nitrate hexahydrate solution was added, confirming the biosynthesis of ZnO nanoparticles. The absorption peaks for UD-ZnO, MC-ZnO, and MK-ZnO NPs were obtained at wavelengths of 308, 319, and 305 nm, respectively (Figure 3). 

The absorption edge consistently moves to a higher energy or lower wavelength when the size of the NPs decreases. Earlier studies have identified the peaks at 289–385 nm as the SPR (surface plasmon resonance) of ZnO nanoparticles. Using the formula below, the energy band gap (Eg) is calculated from the UV–Vis graphs of UD-ZnO, MC-ZnO, and MK-ZnO NPs as 4.0, 3.8, and 4.0 eV, respectively.

Eg = 1240/*λ_max_*eV

The findings showed that ZnO-NPs were successfully synthesized using aqueous extracts of UD (leaves), MC (flowers), and MK (leaves).

#### 3.1.4. Crystallographic Analysis of ZnO-NPs Using X-ray Diffraction

**Figure 4:**
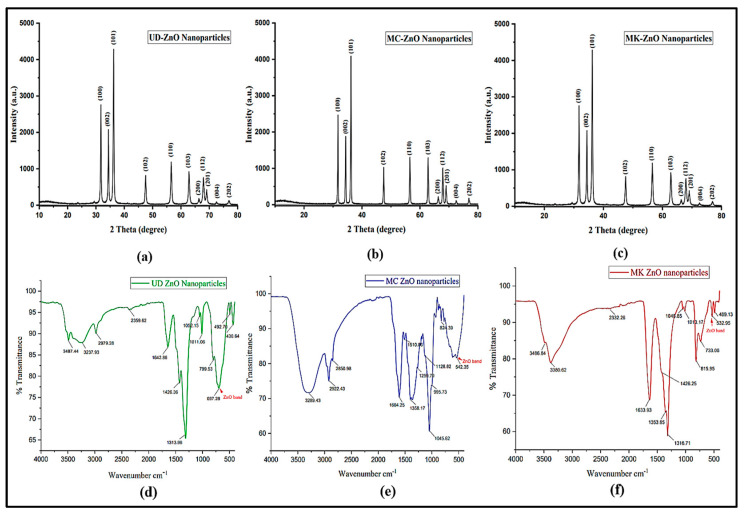
X-ray diffraction (**a**–**c**) and FT-IR absorption spectra (**d**–**f**) with a spectral range of 400–4000 cm^−1^ of *U. dioica* (**a**,**d**), *M. chamomilla* (**b**,**e**), and *M. koenigii* (**c**,**f**) ZnO nanoparticles.

To determine the crystallinity of green-fabricated ZnO-NPs, X-ray diffraction (XRD) was used. ZnO-NPs were subjected to intense XRD rays during the analysis, and these rays passed through the material to reveal information about its physical properties, chemical composition, and crystallographic structure. The diffraction peaks were obtained for 2θ values at 31.7, 34.5, 36.4, 47.8, 56.2, 62.5, 66.2, 67.6, 69.1, 72.9, and 77.2, corresponding to the plane of reflections for the values of (100), (002), (101), (102), (110), (103), (200), (112), (201), (004), and (202), respectively. The results exhibited distinct, strong peaks, which denoted that the green-engineered ZnO nanoparticles are pure and crystalline. The high intensity peak of ZnO-NPs is identified as the characteristic peak of UD-ZnO, MC-ZnO, and MK-ZnO NPs from the XRD pattern (Figure 4a–c) at 101 planes after comparison with the standard powder diffraction card of the JCPDS “Joint Committee on Powder Diffraction Standards”. The purity of the biogenic ZnO-NPs is confirmed since no further diffraction peaks were detected.

The average sizes of the biogenic UD-ZnO, MC-ZnO, and MK-ZnO NPs were 42, 45, and 41 nm, respectively. The particle size decreased when the FWHM value was increased.

#### 3.1.5. Fourier Transform Infrared (FT-IR) Analysis

FT-IR spectra were utilized in the wavelength range of 400–4000 cm^−1^ to determine the functional groups present in the fabrication of ZnO nanoparticles from the aqueous fractions of UD (leaves), MC (flowers), and MK (leaves), as illustrated in Figure 4d–f. The findings of the FTIR study show that the surface of the nanoparticles contains functional groups related to phytoconstituents, namely, glycosides, alkaloids, tannins, reducing sugars, ursolic acid, flavonoids, phenols, and others. In this study, the existence of phenolic and hydroxyl groups was indicated by the broad energy bands between 3500 and 3200 cm^−1^, which are attributed to stretching of O-H group vibrations. These groups are present in the structures of lignin, cellulose, and hemicellulose. The modest intensity peaks in the 2940–2970 cm^−1^ range could be assigned to the stretching C-H alkaline vibrations, as well as other possible molecules. The bands observed at 2300–2370 cm^−1^ could be attributed to -C≡C- stretching. There is a detectable N=O vibration in the range of 1660–1600 cm^−1^. Additionally, weak peaks in this region should indicate the presence of C=C stretching vibration. However, absorption in this area can be a sign of the presence of carbonyl groups. The 1590–1500 cm^−1^ energy bands might be assigned to C=C/amine-NH stretching. The absorption bands between 1455 and 1370 cm^−1^ can be attributed to aromatic ring C-C stretching. The C-O stretching vibrations of the guaiacyl ring can be attributed to the band between 1260 and 1200 cm^−1^. The energy bands identified at 1190–1100 cm^−1^ may correspond to amine stretching. The bands observed between 1096 and 1010 cm^−1^ could be assigned to Si-o-Si stretching protein vibrations. The bands might correspond to secondary amine activity at 867–800 cm^−1^. The deposition of these substances in the synthesis of ZnO-NPs is indicated by the shift of bands to much lower frequencies. In addition, the weak energy bands that were caused by the stretching of the ZnO molecules at 697.28, 542.35, and 532.95 cm^−1^ allowed for greater detection of the formation of UD-ZnO, MC-ZnO, and MK-ZnO NPs, respectively. The area that corresponds to metal–oxygen is between 400 and 600 cm^−1^. 

#### 3.1.6. Analysis of Particle Size Using a Zeta Potentiometer and Dynamic Light Scattering

**Figure 5:**
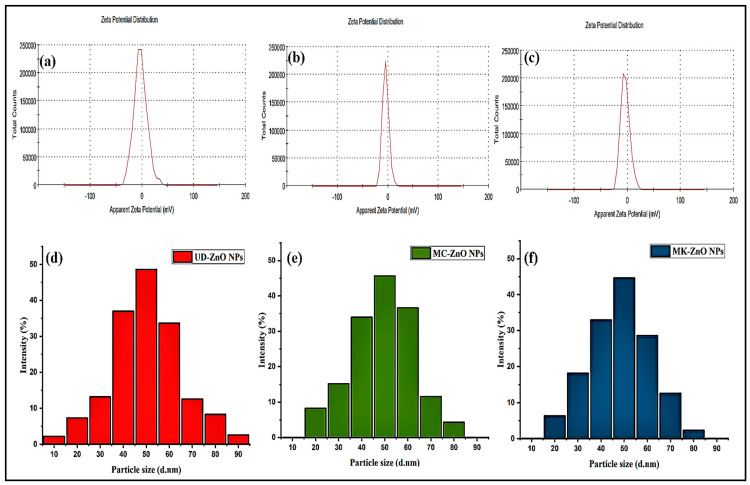
Zeta potential and dynamic light scattering (DLS) of *U. dioica* (**a**,**d**), *M. chamomilla* (**b**,**e**), and *M. koenigii* (**c**,**f**) ZnO nanoparticles.

The size of the synthesized ZnO-NPs was determined using DLS. In this context, the zeta potential is used here to describe the electrochemical charge in the interfacial double layer at the location of the sliding plane in comparison to a site in the bulk fluid far from the interface (Figure 5a–c). The stability of a shape or structure is directly correlated with the zeta potential, also termed the surface potential. 

The distribution curves of UD-ZnO, MC-ZnO, and MK-ZnO NPs are illustrated in Figure 5d–f. It displayed a range of particle sizes, with an average particle size of 43.31 nm and sizes ranging from 10.63 nm to 90.37 nm. Moreover, there was a 45.18 nm gap between the highest and smallest nanoparticle sizes, indicating that the ZnO-NPs were distributed in a restricted range. Additionally, the stability of the nanoparticles was assessed using the zeta potential value, which was found to be −19.2, −17.4, and −18.5 mV for UD-ZnO, MC-ZnO, and MK-ZnO NPs, respectively, indicating reasonably good stability.

The negative value demonstrated the stability of the nanoparticles and prevented their aggregation. The capping effect of the biomolecules found in the aqueous fractions of *U. dioica*, *M. chamomilla*, and *M. koenigii* could be the cause of the negative potential value.

#### 3.1.7. Field Emission Scanning Electron Microscopy

SEM images depicted that ZnO-NPs were formed in a variety of shapes, some of which were spherical and hexagonal at different temperatures, as shown in the SEM images, and others of which were irregular. 

**Figure 6:**
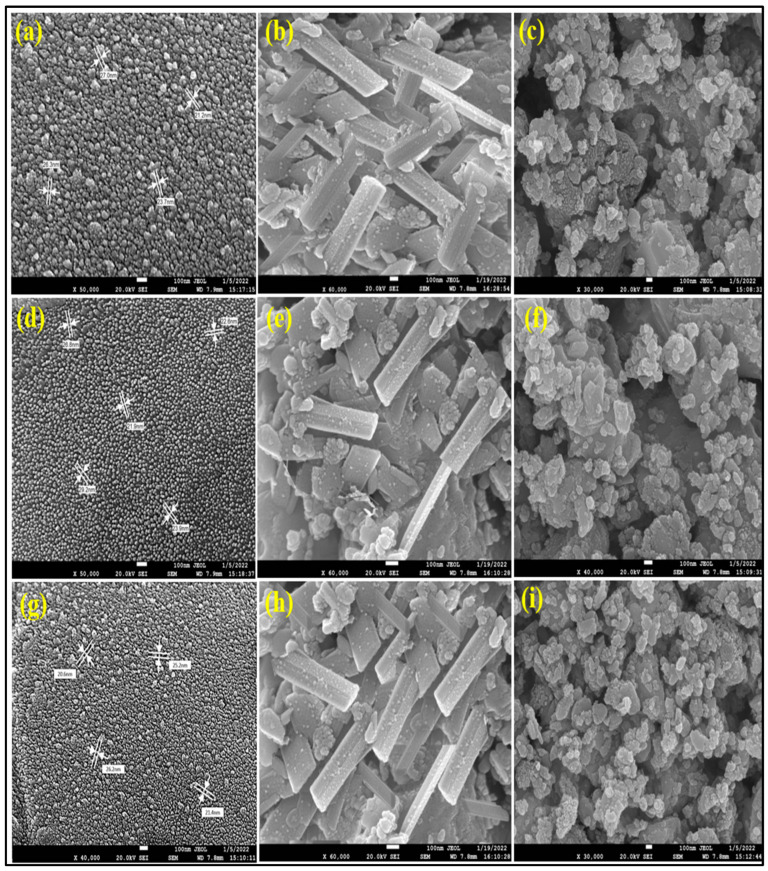
FE-SEM images of green-synthesized ZnO nanoparticles of *U. dioica* (**a**–**c**), *M. chamomilla* (**d**–**f**), and *M. koenigii* (**g**–**i**) at different magnifications and different temperatures. Scale bar = 100 nm.

Different magnifications were used to examine the FE-SEM images. The sizes of the UD-ZnO, MC-ZnO, and MK-ZnO NPs, which range from 29 to 50 nm, are shown in Figure 6. The pH, temperature, and zinc nitrate concentration might have all contributed to the uneven morphology of ZnO nanoparticles.

#### 3.1.8. Energy Dispersive X-ray Spectroscopy

**Figure 7:**
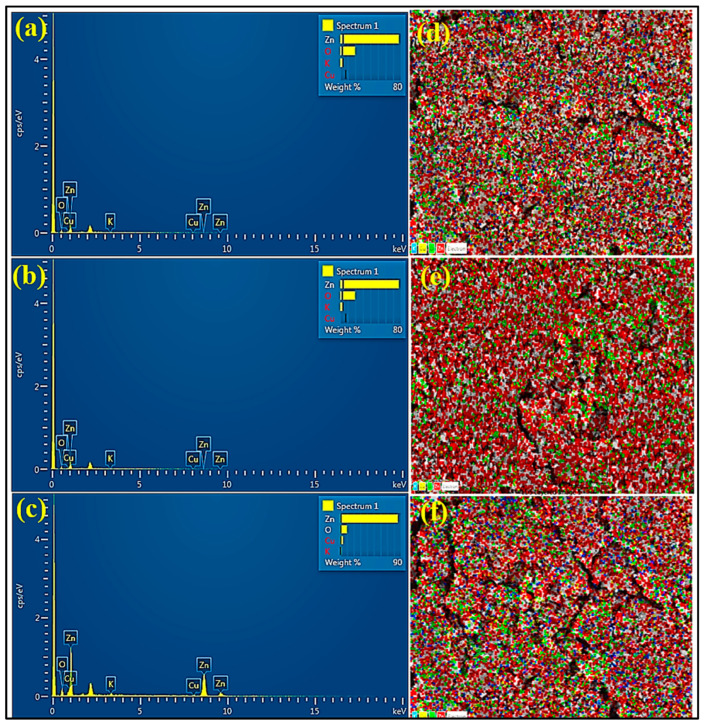
EDX (**a**–**c**) and molecular mapping (**d**–**f**) images of green-synthesized ZnO nanoparticles of *U. dioica* (**a**,**d**), *M. chamomilla* (**b**,**e**), and *M. koenigii* (**c**,**f**) (in molecular mapping red = zinc, green = oxygen, yellow = copper, and blue = potassium).

EDX is an effective technique for determining the elemental composition of UD-ZnO, MC-ZnO, and MK-ZnO NPs. The unique atomic structures of each element provide recognizable peaks on the X-ray spectrum. Other elements on the EDX, such as oxygen and carbon, come from the chemical component of the plant extract that is utilized as a reducing agent. The purity and composition of the green-engineered ZnO-NPs are displayed in the EDX spectrum (Figure 7).

Zn emits a strong signal in the EDX spectrum, while Cu, O, and K emit weak signals. These weak signals are the result of macromolecules such as enzymes, protein, and carbohydrates that are found in the cell wall of the plant fraction emitting X-rays.

#### 3.1.9. Dose-Dependent Antioxidant Potential of Biosynthesized ZnO-NPs Using DPPH and ABTS

The antioxidant and antiradical capacity of green-engineered UD-ZnO, MC-ZnO, and MK-ZnO NPs and their plant extracts is frequently assessed using the DPPH method. Antioxidants are considered to have the ability to donate hydrogen, which helps them scavenge DPPH radicals. When electron/hydrogen donor molecules interact with the organic nitrogen radical, a yellow or brownish radical solution with reduced absorbance is produced. 

**Figure 8:**
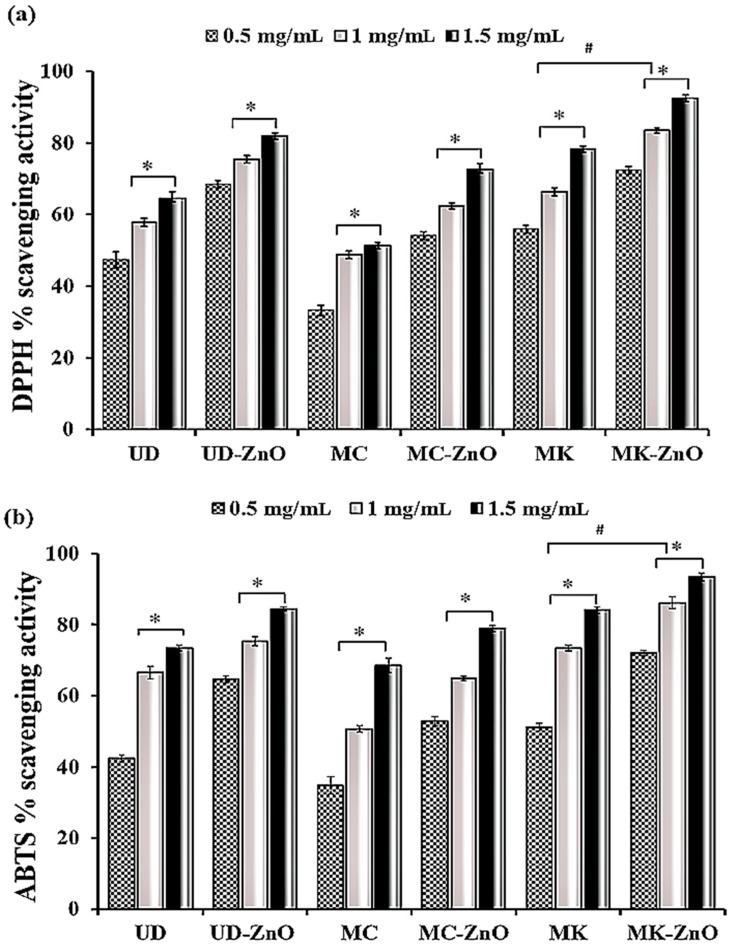
The effect of different concentrations of *Urtica dioica* (UD), *Matricaria chamomilla* (MC), and *Murraya koenigii* (MK) extracts and their synthesized ZnO nanoparticles (UD-ZnO, MC-ZnO, and MK-ZnO NPs) in the DPPH (**a**) and ABTS (**b**) free radical scavenging tests. Data are shown as the mean ± SD for *n* = 3. Statistical significance is ascribed as * *p* < 0.05 (intra group) and # *p* < 0.05 (inter group) compared with 0.5 mg/mL of the respective groups.

The maximum UV–Vis spectral range of DPPH is 515–520 nm. The antioxidant capacity was determined at three different concentrations (0.1, 0.2, and 0.3 mg/mL). In this study, MK-ZnO NPs (72.3–92.5%) exhibited a greater capacity to scavenge DPPH free radicals than the UD-ZnO (68.4–81.9%) and MC-ZnO (54.1–72.7%) nanoparticles, as illustrated in Figure 8a. ZnO-NPs from all three plants showed higher scavenging efficiency when compared to their respective aqueous extracts. The primary determinant of the antioxidant activity of biogenic ZnO-NPs is the redox state of total phenolics, which function as stabilizing agents.

The antioxidant properties of the hydrophilic and hydrophobic bioactive compounds found in the green-fabricated ZnO-NPs were investigated using the unstable free radical ABTS. The maximum UV–Vis spectral range of ABTS is 415 nm. The antioxidant capacity was determined at three different concentrations (0.1, 0.2, and 0.3 mg/mL). These results suggest that biosynthesized MK-ZnO NPs (71.9–93.4%) have stronger antioxidant and antiradical properties and are more effective than UD-ZnO (64.6–84.5%) and MC-ZnO (52.7–78.9%) at scavenging ABTS free radicals. In the samples obtained, it was observed that biosynthesized ZnO-NPs had the maximum radical-scavenging capacity of ABTS, followed by their aqueous extracts, as shown in Figure 8b. The results indicate that when the concentration of biosynthesized ZnO-NPs was enhanced, scavenging activities against both radicals similarly increased, suggesting that the ABTS and DPPH radical scavenging properties were dose-dependent.

#### 3.1.10. EC_50_ Calculation Using Statistical Models

The EC_50_ value is an essential marker for calculating the antioxidant potential of a substance and can be employed to determine the antioxidant strength of various substances. Data from an appropriate curve may be plotted to estimate the EC_50_, or data can be used to run a nonlinear regression with many components. A variety of models may be used to calculate EC_50_. More radical scavenging activity is predicted by lower EC_50_ values.

**Figure 9:**
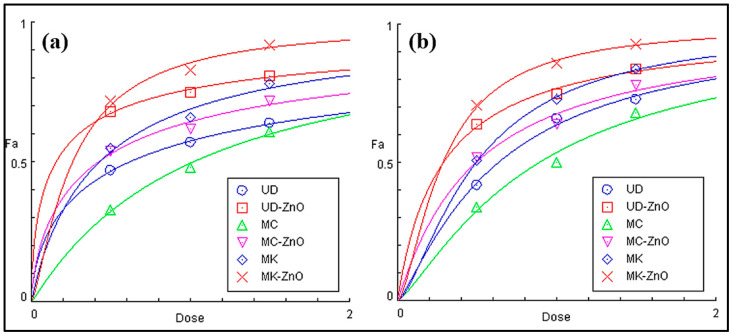
Dose–response curves of the estimated EC_50_ (mg/mL) of *Urtica dioica* (UD), *Matricaria chamomilla* (MC), and *Murraya koenigii* (MK) extracts and their synthesized ZnO nanoparticles (UD-ZnO, MC-ZnO, and MK-ZnO NPs) in the DPPH (**a**) and ABTS (**b**) free radical scavenging tests.

In DPPH, due to their higher total phenolic and flavonoid contents, the biosynthesized MK-ZnO NPs were shown to have stronger antioxidant potential than UD-ZnO and MC-ZnO NPs, which had EC_50_ values of 0.15, 0.25, and 0.41 mg/mL, respectively. A similar pattern was observed in ABTS radicals, MK-ZnO > UD-ZnO > MC-ZnO, with EC_50_ values of 0.21, 0.28, and 0.47 mg/mL, respectively (Figure 9; Table 1).

#### 3.1.11. The Antibacterial Potential of Green-Engineered ZnO-NPs against *S. aureus* and *E. coli*

In the present study, the antimicrobial effect of green-engineered ZnO-NPs was examined against Gram-negative (*Escherichia coli*) and Gram-positive (*Staphylococcus aureus*) bacterial strains following subculturing on Muller–Hinton agar medium.

**Figure 10:**
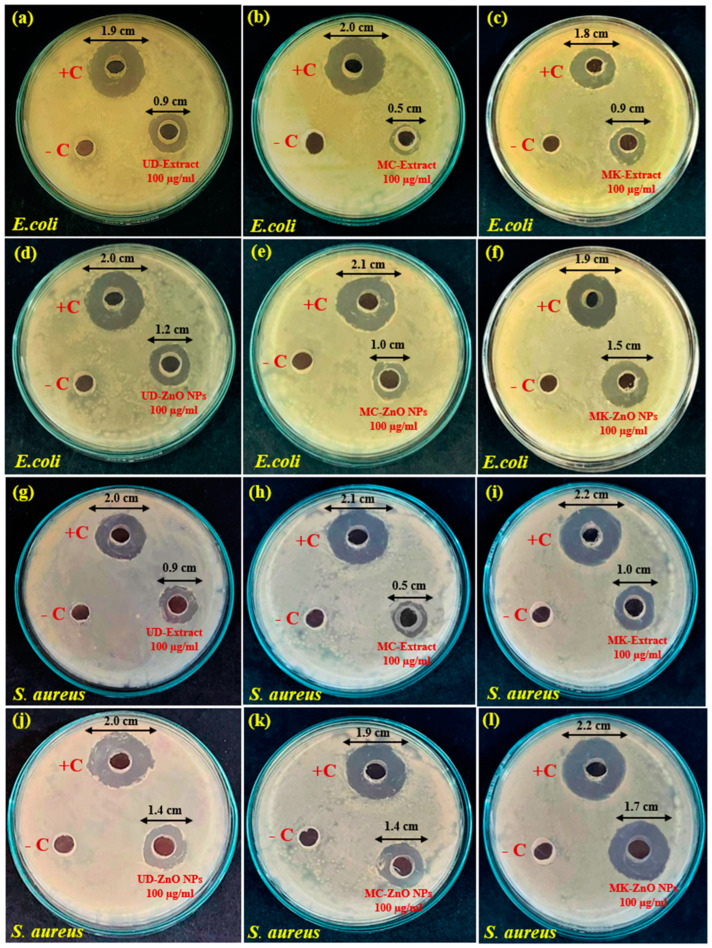
Antibacterial activity of plant extracts (UD, MC, and MK) and their bio synthesized zinc oxide nanoparticles (UD-ZnO, MC-ZnO, and MK-ZnO NPs) against *Escherichia coli* (**a**–**f**) and *Staphylococcus aureus* (**g**–**l**). Note: +C = positive control (chloramphenicol); −C = negative control (deionized water); UD = *Urtica dioica* extract; UD-ZnO = *Urtica dioica* synthesized Zinc Oxide NPs; MC = *Matricaria chamomilla* extract; MC-ZnO = *Matricaria chamomilla* synthesized Zinc Oxide NPs; MK = *Murraya koenigii* extract; MK-ZnO = *Murraya koenigii* synthesized Zinc Oxide NPs.

The maximum inhibition zone of biosynthesized ZnO-NPs in the case of *E. coli* was observed in MK-ZnO NPs (1.5 cm), followed by UD-ZnO NPs (1.2 cm) and MC-ZnO NPs (1.0 cm), as illustrated in Figure 10d–f. The pattern was similar in the case of *S. aureus*, and the highest zone of inhibition was observed for MK-ZnO NPs (1.7 cm), followed by UD-ZnO NPs (1.4 cm) and MC-ZnO NPs (1.4 cm), as shown in Figure 10j–l. 

Due to the existence of peptidoglycan layers, Gram-positive strains have more convergent ZnO-NP penetration into the cell membrane than their negative equivalents. The results suggest that the bacteria are very susceptible to ZnO nanoparticles, which have excellent activity against *E. coli* and *S. aureus*. The plant fractions are rich in amino acids, vitamins, phenols, reducing sugars, and flavonoids, which are essential for binding and capturing ZnO ions. The antibacterial activity measured immediately following synthesis and after storage was found to be equally strong, demonstrating that the activity does not decrease with passing time and confirming the stability of UD-ZnO, MC-ZnO, and MK-ZnO NPs throughout storage processes. The release of zinc ions from ZnO-NPs may potentially contribute to their antibacterial properties. Although the mechanisms for delivering NPs into microbial cells have been identified, more research is still needed to determine exactly how nanoparticles interact with membrane receptors and efflux pumps to initiate antimicrobial activity. There are four ways that NPs can enter a cell, including adhering to the membrane, damaging microbial DNA by producing ROS from Zn ions, interfering with ATP synthesis and DNA replication from ionic forms of nanoparticles, and eventually, forming thiols or phosphates from interactions with nucleic acid and amino acid moieties. However, the primary mechanism by which zinc oxide interacts with bacteria is through interference with both capsule biosynthesis and central carbon metabolism. Higher concentrations of zinc ions in nanoforms impair virulence by lowering the production of hyaluronic acid capsules, which in turn inhibits several crucial enzymes that catabolize glucose and changes the expression of carbon catabolic pathways. The green-fabricated ZnO-NPs can be used for both diagnostic and therapeutic purposes for a variety of pathological diseases because of their promising properties. 

The combination of intracellular ROS generation, membrane damage, zinc ion release, and interaction with biomolecules all contribute to the antibacterial activity of green-engineered ZnO-NPs.

### 3.2. Cellular Assay

#### Trypan Blue Staining in Tissues of Green-Engineered ZnO-NP-Exposed *D. melanogaster*

**Figure 11:**
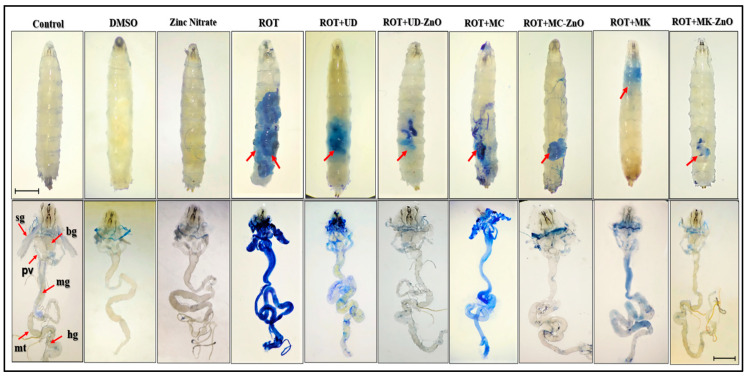
The upper panel shows a dye exclusion test utilizing trypan blue staining in third instar larvae exposed to rotenone and cotreated with UD-ZnO, MC-ZnO, and MK-ZnO. Dissected third instar larvae stained with trypan blue are shown in the lower panel. ROT 500 µM was given to 72-h (±2 h) old *Drosophila melanogaster* (Oregon R^+^) larvae early third instar alone or in combination with UD-ZnO, MC-ZnO, and MK-ZnO for 48 h. Using trypan blue staining, the arrows in the upper panel show cytotoxicities in the whole larvae. “Note: sg = salivary glands, pv = proventriculus, bg = brain ganglia, mt = Malpighian tubules, mg = midgut, and hg = hindgut”. The bar represents 100 µm, ROT = rotenone; UD = *Urtica dioica* extract; UD-ZnO = *Urtica dioica*-synthesized zinc oxide NPs; MC = *Matricaria chamomilla* extract; MC-ZnO = *Matricaria chamomilla*-synthesized zinc oxide NPs; MK = *Murraya koenigii* extract; MK-ZnO = *Murraya koenigii*-synthesized zinc oxide NPs and Arrow represents toxicity.

Trypan blue staining was carried out in the tissues and whole larvae of *Drosophila* to determine whether exposure to rotenone generates any tissue damage (Figure 11). In 96% of the larvae treated with ROT, the whole larvae and their tissues exhibited blue staining (midgut, brain ganglia, gastric caeca, salivary gland, and Malpighian tubules). ROT co-exposed with MK-ZnO NPs demonstrated considerably less blue staining in the tissues mentioned above and the whole larvae when compared to the ROT plus UD-ZnO and ROT plus MC-ZnO NPs groups, respectively.

### 3.3. Biochemical Assays

#### 3.3.1. Biosynthesized ZnO-NPs Enhanced AchE Activity in Rotenone-Exposed *D. melanogaster*

It was observed in this study that the third instar larvae exposed for 24 h to ROT showed a statistically significant (*p* < 0.001) decrease in acetylcholinesterase activity when compared to either DMSO or control, with 68.91% lower AChE levels in the said group. These higher levels of acetylcholinesterase were clearly considerable when compared to ROT-alone-treated groups, with just 20.65% inhibition being seen as opposed to control when ROT and MK-ZnO NPs were co-exposed. Interestingly, there was a considerable improvement in the AChE levels in the ROT plus UD-ZnO and ROT plus MC-ZnO NPs groups (31.85% and 43.75% inhibition, respectively). Plant fractions also demonstrated encouraging effects in the suppression of rotenone-induced acetylcholinesterase inhibition (UD-44.53%, MC-52.03%, and MK-33.79 inhibition).

**Figure 12:**
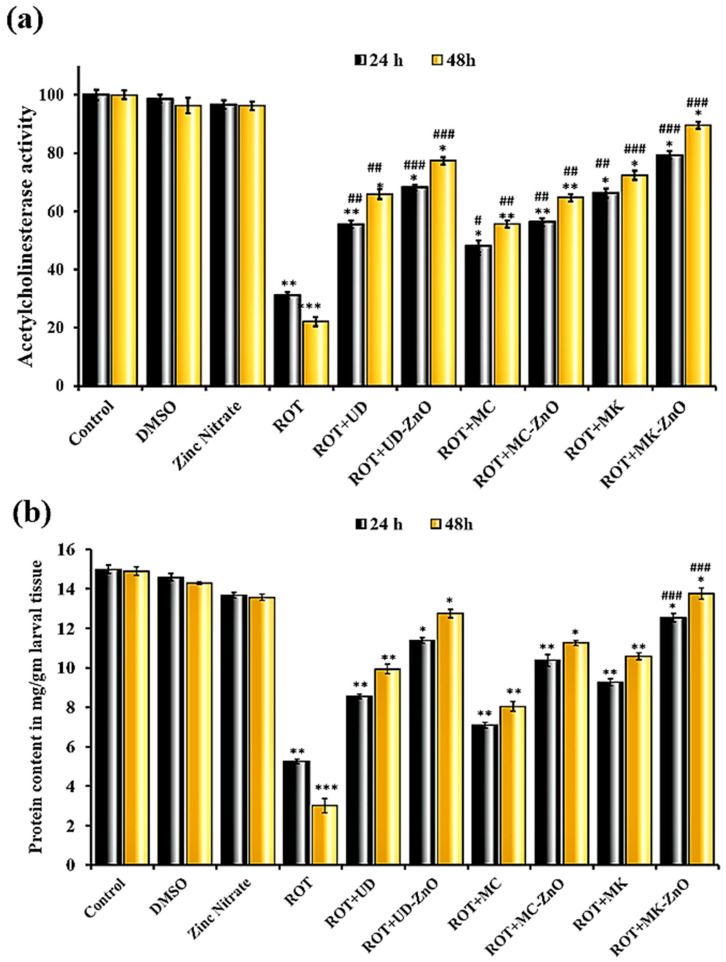
Acetylcholinesterase (**a**) and total protein content (**b**) in the third instar larvae of *D. melanogaster* (Oregon R^+^) exposed to 500 µM ROT alone or in combination with UD, MC, and MK extracts and their synthesized nanoparticles (ZnO-NPs) for 24 and 48 h. “Data represent mean ± SD (*n* = 3); significance ascribed as * *p* < 0.05, ** *p* < 0.01, *** *p* < 0.001 vs. control or DMSO control. ^#^ is ascribed as significance at *p* < 0.05, ^##^ is ascribed as significance at *p* < 0.01, ^###^ *p* < 0.001 as compared with 500 µM rotenone. Note: ROT = Rotenone, UD = *Urtica dioica* extract, UD-ZnO = *Urtica dioica* synthesized Zinc Oxide NPs, MC = *Matricaria chamomilla* extract, MC-ZnO = *Matricaria chamomilla* synthesized Zinc Oxide NPs, MK = *Murraya koenigii* extract, MK-ZnO = *Murraya koenigii* synthesized Zinc Oxide NPs”.

After 48 h, ROT-alone-exposed organisms had the highest amount of AChE inhibition (77.92%) opposed to control/untreated larvae, while the ROT plus MK-ZnO NPs group had the highest degree of rescue from ROT-induced neurotoxicity (10.42%). Additionally, the ROT plus UD-ZnO and ROT plus MC-ZnO groups had considerably greater AChE levels than the ROT-treated group (22.64% and 35.35% inhibition, respectively) as illustrated in Figure 12a. Plant extracts also showed promising results against rotenone-induced acetylcholinesterase inhibition (UD-34.16%, MC-44.46%, and MK-27.64).

#### 3.3.2. Decreased Protein Concentration in Drosophila Exposed to ROT after 24 and 48 h

Rotenone can affect dopaminergic neurons in *Drosophila*. Dopamine-related proteins, such as tyrosine hydroxylase (TH) or dopamine transporters, may be altered in response to rotenone exposure, potentially leading to changes in protein concentration. The total protein concentration in the tissues of third instar larvae of *Drosophila* exposed to ROT (500 µM) decreased statistically significantly (*p* < 0.001). When compared to the control group (14.98 ± 0.211 mg/g), the protein concentration of the larvae in the ROT (6.41 ± 0.258 mg/g) group was lower after 24 h. The highest protein content was observed in ROT co-exposed with MK-ZnO NPs (11.09 ± 0.214 mg/mL), followed by ROT + UD-ZnO NPs (10.08 ± 0.212 mg/g), and the lowest in ROT + MC-ZnO NPs (9.06 ± 0.235 mg/g). Plant extracts such as UD (8.55 ± 0.11 mg/mL), MC (7.09 ± 0.14 mg/mL), and MK (9.27 ± 0.17 mg/mL) also enhanced protein concentration as well. 

After 48 h, the ROT (5.08 ± 0.258 mg/g) exposure has reduced the protein content in the larvae compared to the control group (14.77 ± 0.328 mg/g). ROT co-exposed with MK-ZnO NPs showed an increase in protein concentration (12.9 ± 0.323 mg/g), followed by ROT + UD-ZnO (11.85 ± 0.231 mg/g), and ROT + MC-ZnO NPs (10.65 ± 0.256 mg/g) (Figure 12b). Protein concentration was also increased by plant extracts including UD (9.94 ± 0.24 mg/g), MC (8.04 ± 0.24 mg/g), and MK (10.58 ± 0.17 mg/g). 

### 3.4. Behavioral Assays

#### 3.4.1. Significant Variation in Locomotor Activity of ROT-Treated *Drosophila*

**Figure 13:**
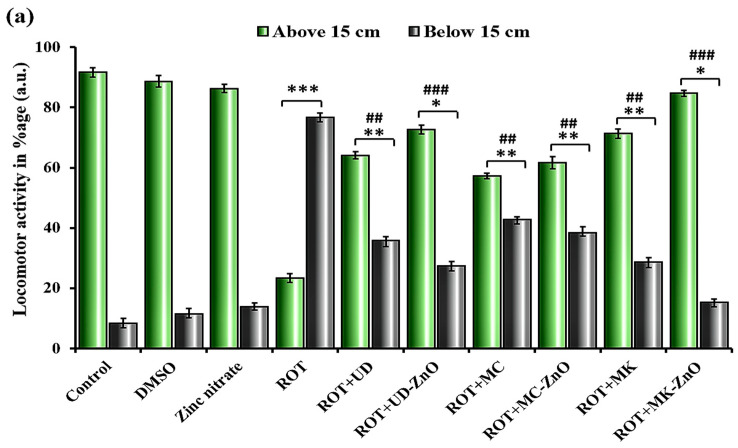

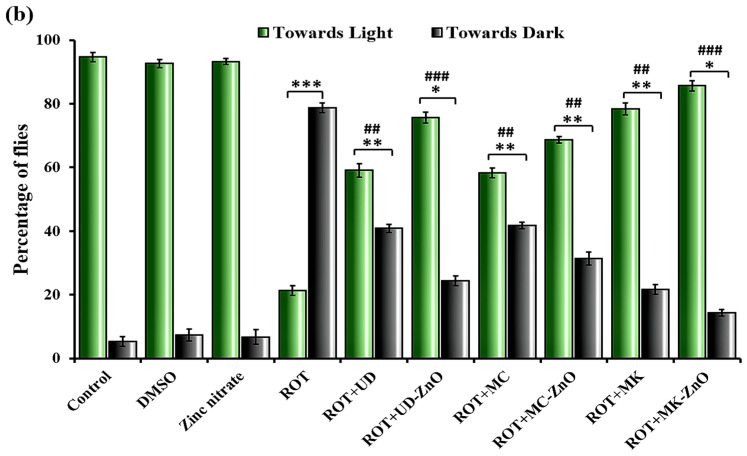
Climbing activity (**a**) and memory ability (**b**) of *Drosophila melanogaster* (Oregon R^+^) flies exposed to ROT (500 µM) alone or in association with UD-ZnO, MC-ZnO, and MK-ZnO for 120 h; “Data represent mean ± SD (*n* = 3); significance ascribed as * *p* < 0.05, ** *p* < 0.01, *** *p* < 0.001 vs. control or DMSO control. ^##^ is ascribed as significance at *p* < 0.01, ^###^ *p* < 0.001 as compared with 500 µM rotenone. Note: ROT = Rotenone, UD = *Urtica dioica* extract, UD-ZnO = *Urtica dioica* synthesized Zinc Oxide NPs, MC = *Matricaria chamomilla* extract, MC-ZnO = *Matricaria chamomilla* synthesized Zinc Oxide NPs, MK = *Murraya koenigii* extract, MK-ZnO = *Murraya koenigii* synthesized Zinc Oxide NPs”.

Rotenone-induced mitochondrial dysfunction has also been associated with oxidative stress and the generation of ROS, which can further contribute to the impairment of locomotor activity in *Drosophila*. The highest climbing potential was shown by the control, DMSO, and zinc nitrate-treated flies in 30 s (only 8.33, 11.41, and 13.47% decrease, respectively). The maximum decrease was detected in ROT-treated *Drosophila* (65.5%), and flies had trouble in climbing the walls of cylinder. Different groups with ROT and plant-mediated ZnO-NPs demonstrate variable degrees of improvement in their climbing abilities. The ability of flies to ascend was increased by all green ZnO-NPs as illustrated in Figure 13a. 

Among the synthesized ZnO-NPs groups, ROT + MK-ZnO NPs (17.62%) exhibited the least reduction, followed by the ROT + UD-ZnO (27.85%) and ROT + MC-ZnO (38.25%) which showed the greatest reduction in climbing activity. The obtained results suggest that plant extracts as well as their synthesized ZnO-NPs enhanced locomotor activity in *Drosophila melanogaster* that had been exposed to rotenone. An unpaired Student’s *t*-test was used to compare the mean ± SEM in order to examine any significant difference. The significance level was determined at *p* < 0.001.

#### 3.4.2. Phyto-Synthesized ZnO-NPs Enhanced Memory in Rotenone-Exposed *D. melanogaster*

Rotenone is known to cause mitochondrial malfunction and oxidative stress, which can impair neuronal function and cognitive processes such as memory. The highest memory ability was shown by the control, DMSO, and zinc nitrate-treated flies (only 5.33, 7.33, and 6.47% reduction, respectively). The maximum decrease was detected in ROT-treated *Drosophila* (78.6%), and flies was observed random movement at the T-point of the apparatus and the flies were slow enough to reach the T-point. The ability of flies to memorize was increased by all green ZnO-NPs as illustrated in Figure 13b. 

Among the synthesized ZnO-NPs groups, ROT + MK-ZnO NPs (14.33%) exhibited the least reduction, followed by the ROT + UD-ZnO (24.33%), and the ROT + MC-ZnO (31.33%) showed the greatest reduction in memory ability. Obtained results suggest that plant extracts as well as their synthesized ZnO-NPs enhanced memory in *Drosophila* melanogaster that had been exposed to rotenone. An unpaired Student’s *t*-test was used to compare the mean ± SEM in order to examine any significant difference. The significance level was determined at *p* < 0.001.

#### 3.4.3. Rotenone Affects Jumping Activity in Drosophila

**Figure 14:**
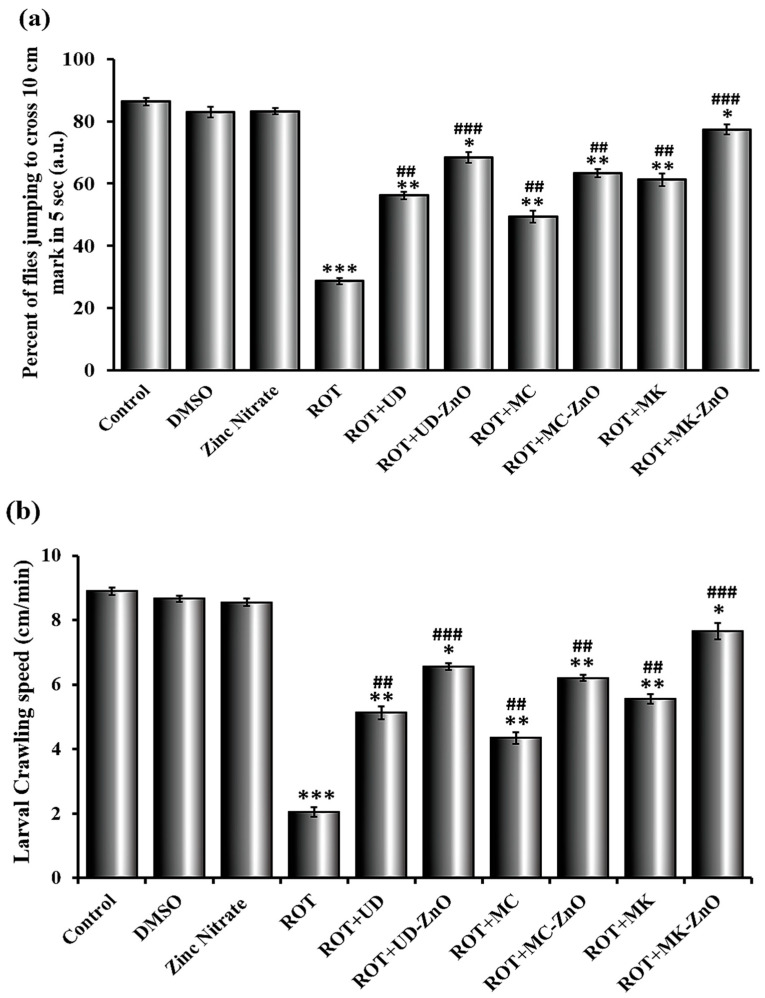
Jumping activity (**a**) and crawling ability (**b**) of *Drosophila melanogaster* (Oregon R^+^) exposed to ROT (500 µM) alone or in association with UD-ZnO, MC-ZnO, and MK-ZnO for 120 h; “Data represent mean ± SD (*n* = 3); significance ascribed as * *p* < 0.05, ** *p* < 0.01, *** *p* < 0.001 vs. control or DMSO control. ^##^ is ascribed as significance at *p* < 0.01, ^###^ *p* < 0.001 as compared with 500 µM rotenone. Note: ROT = Rotenone, UD = *Urtica dioica* extract, UD-ZnO = *Urtica dioica* synthesized Zinc Oxide NPs, MC = *Matricaria chamomilla* extract, MC-ZnO = *Matricaria chamomilla* synthesized Zinc Oxide NPs, MK = *Murraya koenigii* extract, MK-ZnO = *Murraya koenigii* synthesized Zinc Oxide NPs”.

The normal jumping behavior is dependent on the proper functioning of the nervous system and muscle activity, both of which require adequate energy. When compared to control, DMSO, and zinc nitrate (13.67%, 17.02%, and 16.75%), we saw a considerably reduced jumping activity in the *Drosophila* flies treated with ROT (71.4%). Different groups with ROT and green-fabricated ZnO-NPs displayed variable degrees of improvement in their jumping abilities. The ability of flies to ascend was increased by all green ZnO-NPs as illustrated in Figure 14a. 

The lowest reduction is shown by the ROT + MK-ZnO (22.67%), followed by the ROT + UD-ZnO (31.67%), and the maximum reduction is shown by the ROT + MC-ZnO (44.04%). When exposed to rotenone, *Drosophila* exhibit a decrease in jumping activity. This decrease can be attributed to the reduced energy levels caused by the inhibition of complex I. The flies may show a decrease in overall motor activity, including their ability to perform coordinated jumping movements. An unpaired Student’s *t*-test was used to compare the mean ± SEM in order to examine any significant difference. The significance level was determined at *p* < 0.001.

#### 3.4.4. Green ZnO-NPs Elevated Crawling Activity in ROT-Treated Drosophila Larvae

In order to detect any deficits in their locomotor function and consequent behavioral alterations, the crawling pattern of third instar larvae of *Drosophila* was examined. The basic behavior of *Drosophila* larval crawling enables us to examine the involvement of genes in the specification of neurons in the entire organism as well as the functions of those neurons. The control, DMSO, and zinc nitrate-treated larvae had the highest level of crawling activity in 1 min (8.89 ± 0.12, 8.66 ± 0.09, and 8.55 ± 0.98 cm/min, respectively). The largest reduction was seen in *Drosophila* treated with ROT (3.05 ± 0.14 cm/min), where flies had difficulty crawling on petri plates. 

Different ROT and plant-mediated ZnO-NP groups exhibit varying degrees of improvement in their ability to crawl. All green ZnO-NPs improved the ability of larvae to crawl appropriately, as shown in Figure 14b. In this study, it was observed that the crawling ability was highly rescued in ROT + MK-ZnO NPs (7.25 ± 0.25 cm/min), followed by ROT + UD-ZnO (6.76 ± 0.10 cm/min), and ROT + MC-ZnO (5.56 ± 0.05 cm/min) among the synthesized ZnO-NPs groups. The mean and SEM were compared using an unpaired Student’s *t*-test to determine any difference that could be statistically significant. The significance level was determined at *p* < 0.001.

## 4. Discussion

This study demonstrated the antioxidant and antimicrobial properties of biosynthesized UD-ZnO, MC-ZnO, and MK-ZnO nanoparticles as well as rotenone-induced neurological, organismal, and cellular toxicity and its modulation through green-engineered ZnO nanoparticles for the first time in a nontarget organism, *D. melanogaster*.

This study is a continuation of our previous study in which phytoextracts were prepared from all three plants, i.e., UD (*Urtica dioica* leaves), MC (*Matricaria chamomilla* flowers), and MK (*Murraya koenigii* leaves) and their bioactive compounds were evaluated using in vitro biochemical parameters (DPPH, ABTS, TPC, and TFC), UV-Vis, followed by FT-IR and HPLC. Moreover, the amelioration of these herbs against rotenone-induced toxicity in wild-type *Drosophila melanogaster* (Oregon R^+^) at biochemical, cellular, and behavioral levels was reported [14]. 

In this study, pure ZnO-NPs of high grade were synthesized by a green chemistry approach using UD, MC, and MK aqueous extracts as a reducing agent and zinc nitrate hexahydrate as the precursor. The green fabrication mechanism for the generation of zinc oxide nanoparticles based on plant phytochemicals can function in stabilizing and reducing compounds to convert metallic precursors to metallic nanoparticles [55]. Phytoconstituents are antioxidants and nontoxic compounds that can serve as both stabilizing and reducing agents. The major secondary metabolites include methylxanthines, alkaloids, aldehydes, terpenoids, flavonoids, and phenolic acids that are coordinated to the reduction process [56]. These phytoconstituent capping/reducing compounds are present in various plant extracts at varying concentrations. Thus, the synthesis of nanoparticles is significantly influenced by the composition of the plant fraction [57]. The fabrication, stability, and generation of nanoparticles are all dependent on factors such as reaction time, temperature, pH, metal salt concentration, contact time, and phytochemical profile of the plant extract. To stabilize the metal ions after being reduced by plant extracts, they will be encapsulated as an organic coating in three processes, namely, the “activation phase (reduction of metal ions and the nucleation of reduced metal ions are involved), growth phase (contributes to the stability of nanoparticles) and termination phase (NP shapes are determined)” [58]. The activity of secondary metabolites promotes the synthesis of metal oxides for metals such as silver, copper, gold, zinc, titanium, nickel, and iron. Metal ions enter the phase of growth and stabilization through the action of phytochemicals [59].

The absorption peaks for UD-ZnO, MC-ZnO, and MK-ZnO NPs were obtained at wavelengths of 308, 319, and 305 nm, respectively.) Previous study claim that when the size of the NPs decreases, the absorption edge constantly shifts to a higher energy or lower wavelength [60]. Earlier studies have identified the peaks at 289 to 385 nm as the SPR (surface plasmon resonance) of ZnO nanoparticles [28]. The energy band gap is calculated from the UV–Visible graphs of UD-ZnO, MC-ZnO, and MK-ZnO-NPs as 4.0, 3.8, and 4.0 eV, respectively. The mobility of the electronic cloud on the overall structure of the ZnO-NPs may be responsible for the wide absorption band that extends towards longer wavelengths. The plant fraction was also subjected to UV–Vis analysis, and the findings revealed several peaks at various wavelengths between 200 and 350 nm. The plant fractions were found to contain proteins, reducing sugars, and antioxidant molecules. It is crucial to note that these results are strikingly analogous to earlier studies [61]. In general, the considerably quick color shift and sharp absorbance intensity shown in the initial few min of the procedure demonstrate the capabilities of the UD, MC, and MK fractions to produce nanoparticles.

XRD was utilized to evaluate the crystalline structure and phase fraction analysis of biogenic ZnO-NPs. Peak positions of the diffraction pattern were utilized to demonstrate the translational symmetry-size and shape of the unit cell [62]. The diffraction peaks of biogenic ZnO nanoparticles were obtained for 2θ values at 31.7, 34.5, 36.4, 47.8, 56.2, 62.5, 66.2, 67.6, 69.1, 72.9, and 77.2 corresponding to the plane of reflections for the values of (100), (002), (101), (102), (110), (103), (200), (112), (201), (004), and (202), respectively. These results exhibit distinct, strong peaks, which denote that the biogenic ZnO-NPs are pure and crystalline. After comparison with “the standard powder diffraction card of JCPDS”, the high intensity peak of ZnO nanoparticles is recognized as the characteristic peak of UD-ZnO, MC-ZnO, and MK-ZnO nanoparticles from the XRD pattern at 101 planes. The purity of the fabricated ZnO-NPs was confirmed since no further diffraction peaks were detected. Hence, the XRD analysis demonstrated that the metal ions reduced by UD, MC, and MK plant extracts could result in the fabrication of ZnO-NPs with well-defined dimensions. Using the Debye–Scherrer equation, the average sizes of the biosynthesized UD-ZnO, MC-ZnO, and MK-ZnO NPs were determined to be 42, 45, and 41 nm, respectively, which revealed that the nanoparticles had a spherical crystal structure. Equivalent X-ray diffraction patterns were recorded in previous studies for the green synthesis of ZnO nanoparticles [63,64].

FT-IR analysis further confirmed the existence of chemical bonds, organic molecules, and functional groups involved in the conversion of Zn(NO_3_)_2_ to ZnO nanoparticles [65,66]. The findings of the FTIR study show that the surface of the nanoparticles contains phytoconstituents, namely, glycosides, alkaloids, tannins, reducing sugars, ursolic acid, flavonoids, phenols, and others. The existence of phenolic and hydroxyl groups was indicated by the broad energy bands between 3500 and 3200 cm^−1^, which are attributed to O-H group vibrations. The modest intensity peaks in the 2940–2970 cm^−1^ range could be assigned to the stretching C-H alkaline vibrations, as well as other possible molecules. The bands observed at 2300–2370 cm^−1^ may be attributed to -C≡C- stretching. There is a detectable N=O vibration in the 1660–1600 cm^−1^ range. However, absorption in this region can be a sign of the presence of carbonyl groups. The energy bands identified at 1190–1100 cm^−1^ may correspond to amine stretching. The bands observed between 1096 and 1010 cm^−1^ could be attributed to Si-o-Si stretching protein vibrations. The bands might correspond to secondary amine activity at 867–800 cm^−1^. In addition, the weak energy bands that were caused by the stretching of the ZnO molecules at 697.28, 542.35, 532.95 cm^−1^ allowed for greater detection of the generation of UD-ZnO, MC-ZnO, and MK-ZnO NPs, respectively. The area that corresponds to metal–oxygen is between 400 and 600 cm^−1^. Consequently, it can be concluded that either an oxidation or a reduction mechanism is responsible for the major chemical processes involved in the biosynthesis of ZnO-NPs using the aqueous extracts of UD, MC, and MK. Enzymes and phytochemicals found in biological materials contribute to converting metal compounds into nanoparticles. These findings correlate with previously published reports of ZnO nanoparticle synthesis using olive leaf extract [67].

DLS was utilized to measure the size of the fabricated ZnO-NPs. The particle size of the biosynthesized ZnO nanoparticles ranged from 10.63 nm to 90.37 nm, with a mean range of 43.31 nm. The greatest and smallest sizes of the NPs were separated by a gap of 45.18 nm, showing that the distribution of ZnO-NPs was restricted. Furthermore, the zeta potential value was used to evaluate the stability of the nanoparticles. It was discovered to be −19.2, −17.4, and −18.5 mV for UD-ZnO, MC-ZnO, and MK-ZnO NPs, respectively, indicating excellent stability. ZnO-NPs have a negative surface charge because *U. dioica*, *M. chamomilla*, *and M. koenigii* extract compounds have a great affinity for them, increasing their stability and decreasing their inclination to aggregate [68,69].

SEM is a field optical analysis that can evaluate different particle sizes, formations, shapes, densities, and surface morphologies of fabricated NPs at the “nano and micro” sizes (10^−9^ and 10^−6^), respectively [70]. According to the SEM images, some of the ZnO-NPs formed had spherical and hexagonal shapes, while others had irregular shapes. The ZnO-NPs were formed in a variety of shapes, some of which were spherical and hexagonal at different temperatures, as shown in the SEM images, and others of which were irregular. Different magnifications were used to examine the FE-SEM images. The sizes of the UD-ZnO, MC-ZnO, and MK-ZnO nanoparticles ranged from 20 to 50 nm. The pH, temperature, and concentration of Zn(NO_3_)_2_ might have all contributed to the uneven morphology of ZnO nanoparticles [71].

EDX spectroscopy can be utilized to identify the chemical compositions of ZnO-NPs. Each component has a different atomic composition, which results in distinct peaks in the X-ray spectrum. The active ingredient of the plant fraction utilized as a reducing/stabilizing agent is the source of other elements. Zinc emits a strong signal in the EDX spectrum, while Cu, O, and K emit weak signals. These weak signals are the result of macromolecules such as enzymes, protein, and carbohydrates that are present in the cell wall of the plant extract emitting X-rays [72].

The antioxidant properties of green-engineered UD-ZnO, MC-ZnO, and MK-ZnO NPs and their plant extracts were evaluated using ABTS and DPPH free radical scavenging assays [73]. At different concentrations (0.1, 0.2, and 0.3 mg/mL), the antioxidant potential was assessed. In the case of DPPH, MK-ZnO NPs (72.3–92.5%) exhibited a higher capacity to eliminate free radicals than UD-ZnO (68.4–81.9%) and MC-ZnO (54.1–72.7%) NPs. The ABTS test revealed a similar pattern, with biosynthesized MK-ZnO NPs (71.9–93.4%) followed by UD-ZnO (64.6–84.5%) and MC-ZnO (52.7–78.9%) in terms of their ability to scavenge free radicals. ZnO-NPs biosynthesized using extracts of the selected plants have been found to display more intensified antioxidant properties than plant extracts. The results are contradictory since some studies found higher antioxidant activity in ZnO-NPs, while others found the reverse. Considering that a large number of studies have reported both types of results, both outcomes seem possible. This finding suggests that the ABTS and DPPH radical scavenging abilities were dose-dependent; as the concentration of biosynthesized ZnO-NPs increased, the scavenging potential against both radicals also increased [74].

To observe the antibacterial effects of biosynthesized ZnO-NPs, an experiment on *E. coli* and *S. aureus* was carried out. The zone of inhibition of UD-ZnO, MC-ZnO, and MK-ZnO NPs and their efficiency in comparison to an antibiotic (chloramphenicol) were the major considerations. The maximum inhibition zone of biosynthesized ZnO-NPs in the case of *E. coli* was observed in MK-ZnO NPs (1.5 cm), followed by UD-ZnO NPs (1.2 cm) and MC-ZnO NPs (1.0 cm). A similar pattern was observed in the case of *S. aureus*, and the highest inhibition zone was observed for MK-ZnO NPs (1.7 cm), followed by UD-ZnO NPs (1.4 cm), and MC-ZnO NPs (1.4 cm). Although the mechanisms for delivering NPs into microbial cells have been identified, more research is still needed to determine exactly how nanoparticles interact with membrane receptors and efflux pumps to initiate antimicrobial activity [75,76]. There are four ways that NPs can enter a cell, including adhering to the membrane, damaging microbial DNA by producing ROS from Zn ions, interfering with DNA replication and ATP synthesis from ionic forms of nanoparticles, and eventually forming thiols or phosphates from interactions with nucleic acid and amino acid moieties. However, the primary mechanism by which zinc oxide interacts with bacteria is through interference with both capsule biosynthesis and central carbon metabolism. Higher concentrations of zinc ions in nanoforms impair virulence by lowering the production of hyaluronic acid capsules, which in turn inhibits several crucial enzymes that catabolize glucose and change the expression of carbon catabolic pathways [77]. The destructive activity of UD-ZnO, MC-ZnO, and MK-ZnO NPs at minimum inhibitory levels for bacteria is most likely caused by their ability to damage cell membrane structures and permeability barriers with loss of chemiosmotic control. The finding correlates with the results that have previously been published [78].

Using a dye exclusion (trypan blue) test, the cytotoxic effects of ROT and the alleviation of cytotoxicity by the administration of biosynthesized ZnO-NPs in tissues and whole larvae of *Drosophila* were detected. In 96% of the larvae treated with ROT, the whole larva and its tissues exhibited blue staining (salivary gland, midgut, brain ganglia, Malpighian tubules, and gastric caeca). ROT co-exposed with MK-ZnO NPs demonstrated significantly less blue staining in the tissues mentioned above and the whole larvae when compared to the ROT plus UD-ZnO and ROT plus MC-ZnO NPs groups, respectively. This conclusion is validated by a previous investigation that showed that plant biomolecules significantly enhanced cell viability and attenuated DNA damage [79,80]. Biogenic ZnO-NPs are protective due to the activation of antioxidant defense mechanisms or the presence of bioactive molecules that quench free radicals.

Biochemical studies were conducted after 24 and 48 h of treatment to apprehend the adverse effects of rotenone, which elevated cellular oxidant levels and enabled proteins to undergo post-translational oxidative alterations. After 24 h, the total protein concentration in the larval tissues (third instar) of *Drosophila* exposed to ROT (500 µM) decreased significantly (*p* < 0.001) compared to the control and vehicular control (DMSO) groups. The highest protein content was determined in ROT co-exposed with MK-ZnO, followed by ROT plus UD-ZnO, and the least in ROT plus MC-ZnO. A similar pattern was observed after 48 h, and ROT exposure reduced the total protein concentration in the larvae compared to the untreated/control group. ROT co-exposed with MK-ZnO NPs showed an increase in protein concentration, followed by ROT plus UD-ZnO and ROT plus MC-ZnO NPs. This outcome is consistent with earlier studies that showed that pesticides decreased the protein concentration in organisms [81]. The secondary metabolites found in plants that function as free radical scavengers [82], especially against oxygen radicals, and inhibit SH-group oxidation could be the protective mechanism of plant-derived ZnO-NPs.

Acetylcholinesterase is a cholinergic system enzyme that governs physiological functions such as movement and memory [83]. By hydrolyzing acetylcholine into acetate and choline, it inhibits cholinergic neurotransmission across synapses [84]. In this experiment, it was observed that when the *Drosophila* larvae exposed to rotenone for 24 h showed a considerably significant (*p* < 0.001) decrease in acetylcholinesterase activity compared to either DMSO or control, they had ~66.31% reduced AChE levels. The level of AChE was enhanced when ROT was co-administered with MK-ZnO NPs, and only 24.65% inhibition was evident. Comparing the ROT plus UD-ZnO and ROT plus MC-ZnO NPs groups to the control, the improvement in AChE levels was less prominent in both groups. After 48 h, organisms exposed to ROT alone had the highest amount of AChE inhibition compared to control/untreated larvae, and the greatest level of rescue from rotenone-induced neurotoxicity was observed in the ROT plus MK-ZnO NP group. Additionally, the ROT plus UD-ZnO and ROT plus MC-ZnO groups had significantly greater AChE levels than the ROT-treated group. The findings revealed that AChE inhibition in rotenone-exposed organisms and biosynthesized ZnO-NPs contributes to restoring AChE levels, which is consistent with earlier studies [85].

“The behaviour of an organism reflects its usual physiological function”. In this context, an organism’s behaviour of climbing and jumping reflects its physiological condition. Therefore, a higher prevalence of locomotor impairments as assessed by the climbing test could point to rotenone-induced neurotoxicity. Interestingly, MK-ZnO > UD-ZnO > MC-ZnO nanoparticles effectively protected flies from deteriorating neuromuscular dysfunctions, indicating that they might be able to defend by replenishing the dopaminergic pool at the mitochondrial level. The considerable reduction in jumping behavior in exposed organisms, preceded by a suppression of AChE activity, demonstrated the pesticide’s detrimental consequences on the organism [86]. AChE activity suppression has previously been associated with diminished locomotor function. The maximum rescue was demonstrated by ROT plus MK-ZnO, followed by ROT plus UD-ZnO and ROT plus MC-ZnO NPs. The observations support previous studies that plant nanoparticles have the capacity to biosynthesize several antioxidants (nonenzymatic) that can decrease ROS-induced oxidative damage [87,88].

Rotenone exposure has been associated with impaired memory function in various organisms. Memory deficits can manifest as difficulty in learning new tasks, impaired spatial memory, or reduced ability to recall previously learned information. In this observation, the highest memory ability was shown by the control, DMSO, and zinc nitrate-treated flies. The maximum decrease was detected in ROT-treated *Drosophila*, and flies were observed randomly moving at the T-point of the apparatus and the flies were slow enough to reach the T-point. The ability of flies to memorize was increased by all green ZnO-NPs, as illustrated in Figure 13b. Among the synthesized ZnO-NPs groups, ROT plus MK-ZnO NPs exhibited the least reduction, followed by the ROT plus UD-ZnO and ROT plus MC-ZnO showed the greatest reduction in memory ability. Obtained results suggests that plant extracts as well as their synthesized ZnO-NPs enhanced memory in *Drosophila melanogaster* that had been exposed to rotenone. Plant-synthesized ZnO-NPs with antioxidant properties can scavenge harmful reactive oxygen species and reduce oxidative damage, potentially preserving neuronal health and supporting memory processes [89]. The basic behavior of *Drosophila* larvae crawling enables us to examine the involvement of genes in the specification of neurons in the entire organism as well as the functions of those neurons [90]. The control and DMSO-treated larvae had the highest level of crawling activity in 1 min. The largest reduction was observed in *Drosophila* larvae treated with ROT, which had difficulty crawling on petri plates. All plant-derived ZnO-NPs improved the ability of larvae to crawl appropriately. In this study, it was observed that the crawling ability was significantly rescued in ROT plus MK-ZnO, then by ROT plus UD-ZnO, and ROT plus MC-ZnO among the synthesized ZnO-NPs groups [91,92].

The functionalization of biogenic ZnO-NPs with specific biomolecules enables them to act as carriers for drugs, genes, or other therapeutic agents. This targeted delivery approach can improve drug efficacy and minimize side effects [93]. *Drosophila* offers several advantages as an in vivo model for nanomaterial safety assessment; its use provides a comprehensive understanding of the potential risks and mechanism of action associated with nanomaterial exposure [94]. The method of synthesis played a major role towards the toxic potential of ZnO nanoparticles. The green ZnO nanoparticles due to their “green route” of synthesis utilized phytoconstituents as capping agent, which due to their antioxidant properties posed lesser toxicity than pure chemical ZnO nanoparticles as evidenced via cell viability and mortality rate assays [95]. These nanoparticles are more compatible with biological systems, making them suitable for various applications where interactions with living organisms occur [96,97].

Altogether, the present work showed the comparative potency of biosynthesized ZnO-NPs against microorganisms (*E. coli* and *S. aureus*), in addition to the nontarget organism *Drosophila*, at neuronal, organismal, and cellular levels. The synthesized ZnO nanoparticles were analyzed using SEM, UV–Vis, XRD, DLS, EDX, FT-IR, and zeta potential techniques. The bio-fabricated ZnO-NPs using extracts of the selected plants have been found to show more intensified antioxidant and antibacterial properties compared to plant extracts. Short-term dietary administration of UD-ZnO, MC-ZnO, and MK-ZnO NPs to *D. melanogaster* has the potential to reduce ROT-induced oxidative stress, improve locomotion, and restore AChE levels, providing evidence of their neuroprotective properties. The limitations of conventional drug administration are dramatically improved by the green synthesis of ZnO-NPs with drug targeting, sustained release, and significantly increased bioavailability of drugs. More investigation is required to elucidate and mediate the mechanisms by which the blood–brain barrier is crossed, as well as to improve the efficiency of nanotechnology-based brain delivery techniques.

## 5. Conclusions

This study demonstrated that pure ZnO-NPs of excellent quality were fabricated using zinc nitrate hexahydrate as the precursor and plant extracts (UD, MC, and MK) as the reducing agent. The plant-mediated process was determined to be environmentally benign and capable of generating NPs with fewer chemicals than traditional methods. Their optical and structural properties were analyzed through FT-IR, UV–Vis, XRD, DLS, EDS, SEM, zeta size and potential to determine the agglomerated crystalline and hexagonal wurtzite structure with an average diameter of 20–40 nm. Nano-therapeutically, the administration of biogenic ZnO-NPs to *Drosophila* statistically significantly reduced oxidative stress because of its antioxidative properties and ability to regulate antioxidant defenses. Therefore, the application of nanotechnology to produce non-invasive drug delivery systems may result in the development of innovative and enhanced formulations to facilitate the delivery of therapeutic substances across the blood–brain barrier.

## Figures and Tables

**Table 1:** Predicted EC_50_ (mg/mL) of biosynthesized ZnO and its extracts as determined by the various models using DPPH and ABTS tests.

S. No.	Drug	EC_50_ (mg/mL) of DPPH	EC_50_ (mg/mL) of ABTS
1.	Ascorbic Acid	0.4 ± 0.02	0.11 ± 0.05
2.	UD	0.61 ± 0.12	0.63 ± 0.11
3.	UD-ZnO	0.25 ± 0.18	0.28 ± 0.08
4.	MC	1.01 ± 0.47	0.88 ± 0.10
5.	MC-ZnO	0.41 ± 0.21	0.47 ± 0.05
6.	MK	0.42 ± 0.14	0.48 ± 0.17
7.	MK-ZnO	0.15 ± 0.03	0.21 ± 0.07

## Data Availability

Data are contained within the article.
